# Extracellular vesicles from amyloid-β exposed cell cultures induce severe dysfunction in cortical neurons

**DOI:** 10.1038/s41598-020-72355-2

**Published:** 2020-11-12

**Authors:** Chiara Beretta, Elisabeth Nikitidou, Linn Streubel-Gallasch, Martin Ingelsson, Dag Sehlin, Anna Erlandsson

**Affiliations:** Department of Public Health and Caring Sciences, Molecular Geriatrics, Rudbeck Laboratory, Uppsala University, 751 85 Uppsala, Sweden

**Keywords:** Cell death in the nervous system, Cellular neuroscience, Diseases of the nervous system, Glial biology

## Abstract

Alzheimer’s disease (AD) is characterized by a substantial loss of neurons and synapses throughout the brain. The exact mechanism behind the neurodegeneration is still unclear, but recent data suggests that spreading of amyloid-β (Aβ) pathology via extracellular vesicles (EVs) may contribute to disease progression. We have previously shown that an incomplete degradation of Aβ_42_ protofibrils by astrocytes results in the release of EVs containing neurotoxic Aβ. Here, we describe the cellular mechanisms behind EV-associated neurotoxicity in detail. EVs were isolated from untreated and Aβ_42_ protofibril exposed neuroglial co-cultures, consisting mainly of astrocytes. The EVs were added to cortical neurons for 2 or 4 days and the neurodegenerative processes were followed with immunocytochemistry, time-lapse imaging and transmission electron microscopy (TEM). Addition of EVs from Aβ_42_ protofibril exposed co-cultures resulted in synaptic loss, severe mitochondrial impairment and apoptosis. TEM analysis demonstrated that the EVs induced axonal swelling and vacuolization of the neuronal cell bodies. Interestingly, EV exposed neurons also displayed pathological lamellar bodies of cholesterol deposits in lysosomal compartments. Taken together, our data show that the secretion of EVs from Aβ exposed cells induces neuronal dysfunction in several ways, indicating a central role for EVs in the progression of Aβ-induced pathology.

## Introduction

Alzheimer’s disease (AD) develops over decades and is the prevalent causal factor of dementia among the elderly^1^. The main characteristics of AD is the formation of amyloid-β (Aβ) plaques and neurofibrillary tangles, as well as pronounced inflammation. Based on the amyloid cascade hypothesis, accumulation of Aβ is the primary pathological event of AD, which in turn triggers inflammation and the appearance of neurofibrillary tangles, eventually leading to severe neuronal dysfunction^2^. Accumulating evidence indicate that the widespread neurodegeneration in the AD brain is a result of soluble Aβ aggregates, such as protofibrils, rather than insoluble Aβ fibrils^3–5^. However, the exact cellular and molecular mechanisms behind the Aβ-induced neuronal cell death remain elusive.

Since the majority of patients with sporadic AD do not display an increase in Aβ production, insufficient lysosomal degradation of Aβ has been suggested as a possible disease cause^6,7^. In line with such hypothesis, the cells’ ineffectiveness to clear protein aggregates is known to result in Aβ deposits as dense bodies, multivesicular bodies (MVBs) and autophagic vacuoles^8–10^. Moreover, intracellular Aβ accumulation may cause intercellular spreading of Aβ pathology via cell-to-cell contacts and secretion of extracellular vesicles (EVs)^8,11^. There are different forms of EVs, including larger microvesicles (MVs), that originate and bud directly from the plasma membrane and smaller exosomes, that are generated by invagination of the membrane of MVBs and released by exocytosis^12^. However, currently there are no consensus criteria to define different types of EVs, as they often overlap in size and share some surface markers^13^. EVs can be isolated by ultracentrifugation from various fluids, including cell culture medium, plasma, cerebrospinal fluid and urine, and constitute an important form of intercellular communication by delivering proteins, lipids, nucleic acids and sugar molecules to recipient cells through endocytosis^14–17^. Several studies have demonstrated that EVs can carry pathological proteins associated with neurodegenerative diseases, including aggregated forms of Aβ^18^, but their exact role in the disease process remain to be uncovered.

We have previously investigated the cellular responses of synthetic Aβ_42_ protofibrils in a mixed co-culture of astrocytes, neurons and oligodendrocytes. Interestingly, we found that the astrocytes rapidly engulf large amounts of Aβ_42_ protofibrils, followed by storage rather than degradation of the ingested material^8^. The ineffective degradation of Aβ results in astrocytic endosomal/lysosomal defects and secondary neuronal cell death, due to the secretion of neurotoxic EVs. Our previous investigations of the EV content demonstrated that EVs isolated from Aβ_42_ protofibril exposed cultures contain N-terminally truncated forms of Aβ_42_ and increased levels of apolipoprotein E (apoE)^8,19^. Truncated forms of Aβ are present in both intracellular and extracellular deposits in the AD brain^20^ and are known to be more resistant to degradation, more prone to aggregate and more toxic than full-length Aβ^21^. However, exactly how the toxic EVs affect the neurons was not examined. In this follow-up study, we have investigated the mechanisms behind EV-mediated neurotoxicity in detail.

## Materials and methods

### Synthetic Aβ_42_ protofibrils

Aβ_42_ protofibrils were produced according to a well-established protocol^22–24^. In short, synthetic Aβ_42_ peptides were dissolved in 10 mM NaOH, mixed with phosphate-buffered saline (PBS) to a concentration of 443 µM (2 mg/ml) and incubated at 37 °C for 30 min (unlabeled Aβ_42_, Innovagen or American Peptide Company Inc) or 45 min (Fluorescent HiLyte Fluor 555-labeled Aβ_42_, Anaspec Inc). The sample was then centrifuged for 5 min at 17,900×*g* at 4 °C to remove any insoluble aggregates, followed by 1:4 dilution of supernatant in sterile PBS to a final concentration of 0.5 mg/ml. The protofibril specific ELISA, based on mAb158^22^, was used to confirm the Aβ_42_ protofibril concentration. For transmission electron microscopy (TEM), Aβ_42_ protofibrils diluted 1:10 in PBS were dropped onto carbon coated 300-mesh copper grids, negatively stained with 1% Uranyl acetate for 5 min and air dried. The samples were analyzed using a Hitachi H-7100 transmission electron microscope. A careful characterization of the Aβ_42_ protofibrils has been performed previously, using HPLC-SEC, Thioflavin T staining, different ELISAs, in addition to TEM^8,22–24^.

### Animals

All experiments were approved by the Uppsala County Animal Ethics Board (Ethical permit number: C75/13, valid until 2018-06-28 and Ethical permit number: 5.8.18-08472/18, valid until 2023-05-31), following the rules and regulations of the Swedish Animal Welfare Agency, in compliance with the European Communities Council Directive of 22 September 2010 (2010/63/EU). The mice were housed in a 12 h dark–light cycle in an enriched environment and had ad libitum access to food and water.

### Co-cultures of neurons and glia

Cerebral cortices were dissected from C57/BL6 E14 mouse embryos in Hank’s buffered salt solution (HBSS), supplemented with 100 U/ml penicillin and 100 µg/ml streptomycin and 8 mM Hepes buffer (all from Invitrogen). The cortices were centrifuged for 3 min at 150×*g* and the pellet was resuspended in HBSS. Any remaining blood vessels were let to sediment for 10 min at room temperature (RT) and the supernatant was centrifuged for 5 min at 150×*g*. The pellet was resuspended in proliferation medium; DMEM/F12 with added 1 × B27 supplement, 100 U/ml penicillin, 100 µg/ml streptomycin, 8 mM Hepes buffer, 10 ng/ml basic fibroblast growth factor (bFGF) (all from Invitrogen) and 20 ng/ml epidermal growth factor (EGF, Corning). The cells were expanded as neurospheres in non-treated cell culture flasks and passaged every second to third day, by dissociation in HBSS and resuspension in proliferation medium. Prior to experiments, the cells were plated on Poly-L-Ornithine (Sigma-Aldrich) and laminin (Invitrogen) coated coverslips or culture dishes in proliferation medium at a seeding density of 32,000 cells/cm^2^. The following day, the proliferation medium was replaced with differentiation medium (without bFGF and EGF) to initiate differentiation to a mixed population of 75 ± 8% astrocytes, 25 ± 8% neurons and 5 ± 3% oligodendrocytes^8^. During the 7-day differentiation period, the medium was changed every second to third day. Only neurospheres from passage 2–4 were used for experiments.

### Neuronal cell cultures

Dissection and dissociation of C57/BL6 E14-E16 cerebral mouse cortices were performed as described above. The pellet was resuspended in neurobasal medium, supplemented with 1 × B27 supplement, 100 U/ml penicillin, 100 µg/ml streptomycin and 2 mM L-glutamine (all from Invitrogen). The neurons were cultured as a monolayer at a concentration of 25,000 cells/cm^2^ on Poly-l-Ornithine (Sigma-Aldrich) and laminin (Invitrogen) coated coverslips, electron microscopy dishes or cell culture dishes for 12 days in vitro (DIV12). The medium was fully replaced with fresh medium at DIV1, after which only half of the medium was replaced every second or third day.

### Aβ_42_ protofibril exposure of co-cultures

Differentiated co-cultures were exposed to 0.1 µM Aβ_42_ protofibrils in differentiation medium for 24 h. Control cultures received fresh medium without Aβ_42_ protofibrils. No further aggregation of the protofibrils was noted in the medium during the 24 h exposure time^8^. After 24 h of exposure, cells were washed 3 times with medium and thereafter cultured in Aβ_42_ protofibril-free medium for 6 consecutive days (day 6), at which all medium was collected and stored at 4 °C. Fresh Aβ-free medium was added and the cultures were incubated for 6 additional days prior to the next medium collection at day 12.

### Isolation and addition of EVs to neuronal cultures

The conditioned co-culture medium from day 6 was pooled with the equivalent medium from day 12 prior to ultracentrifugation. The pooled medium samples for each treatment and cell culture batch were centrifuged at 300×*g* for 5 min to remove any free-floating cells, followed by another centrifugation at 2,000×*g* for 10 min to remove any remaining cell debris. The supernatants were collected and transferred to ultracentrifuge tubes and centrifuged at 135,000×*g* at 4 °C for 1.5 h to isolate EVs, including larger MVs and exosomes. The vesicle pellets were resuspended in either PBS for TEM analysis or neurobasal medium, supplemented with penicillin, streptomycin, L-glutamine and B27 supplement, for treatment of neuronal cells. DIV12 neuronal cultures were incubated for 2 or 4 days with the EVs from untreated co-cultures or co-cultures that had been exposed to Aβ_42_ protofibrils. Parallel neuronal cultures received medium with or without 0.1 µM Aβ_42_ protofibrils, but no EVs.

### Immunocytochemistry and labeling

Cells were fixed with 4% paraformaldehyde for 15 min at RT and washed 3 times with PBS. For the cholesterol staining, coverslips were incubated with filipin III (1:100 in cholesterol detection buffer, Cholesterol Assay kit, Abcam) for 1 h in the dark at RT. Prior to antibody incubation, the cells were permeabilized and blocked with 0.1% Triton X-100 with 5% normal goat serum (NGS) in PBS for 30 min at RT. Primary antibodies were diluted in 0.1% Triton X-100 with 0.5% NGS in PBS and the coverslips were incubated with the antibody solution for 1–4 h at RT. The primary antibodies used were: rabbit anti-Glial Fibrillary Acidic Protein (GFAP, 1:400, DAKO), mouse anti-βIII tubulin (1:200, Covance), mouse anti-2′,3′-Cyclic-nucleotide 3′-phosphodiesterase (CNPase, 1:500, Sigma-Aldrich), rabbit anti-synaptophysin (1:50, Abcam), rabbit anti-lysosomal-associated membrane protein 1 (LAMP1, 1:100, Abcam), mouse anti-Aβ 4G8 (1:200, Biolegend). The coverslips were washed 3 times with 1 × PBS and then incubated with secondary antibodies, also diluted in 0.1% Triton X-100 with 0.5% NGS in PBS for 45 min at 37 °C. The secondary antibodies used were AlexaFluor 488, 555 or 350 against mouse or rabbit (1:200, Molecular Probes). The coverslips were washed 3 times with PBS and mounted on microscope slides using EverBrite hard-set medium with or without DAPI (Biotium) or Vectashield hard-set mounting medium with DAPI (DAKO). For the cholesterol stainings, Triton X-100 was not included in the blocking solution or antibody dilution. Terminal deoxynucleotidyl transferase dUTP nick end labeling (TUNEL, Roche biochemicals) assay was performed according to the manufacturer’s instructions (protocol 10, to quantify apoptotic cells). For visualization of mitochondria, the neuronal cultures were transfected with CellLight Mitochondria-GFP (Bacman 2.0, ThermoFisher Scientific). The reagent was added in the neurobasal medium 3 days after the addition of EVs according to the manufacturer’s instructions and the neuronal cultures were incubated for 24 h prior to fixation.

### Time-lapse microscopy

Time-lapse experiments were performed at 37 °C in humidified 5% CO_2_ in air, using a Nikon Biostation IM Live Cell Recorder (Nikon). The cells were cultured at a concentration of 25,000 cells/cm^2^, in time-lapse culture dishes (VWR) and pictures were taken every 10th min for up to 48 h^8^.

### Transmission electron microscopy

Transmission electron microscopy was performed as described previously^8,19^.

#### EVs

EV samples were added onto a formvar-coated 200-mesh grid (Oxford 11 Instruments) and incubated for 45 min. The grid was dried, and 1% uranyl acetate was added to the grid and incubated for 10 s. The grid was left to dry for at least 15 min before visualization in a Tecnai G2 transmission electron microscope (TEM, FEI company) with an ORIUS SC200 CCD camera and Gatan Digital Micrograph software (both from Gatan Inc.).

#### Cells

The neuronal cultures were fixed in 2.5% glutaraldehyde and 1% paraformaldehyde. The cells were then rinsed with 0.15 M sodium cacodylate (pH 7.2–7.4) for 10 min and incubated in fresh 1% osmium tetraoxide in 0.1 M sodium cacodylate for 1 h at RT. After incubation, the sodium cacodylate was rinsed away to dehydrate the dishes with 70% ethanol for 30 min, 95% ethanol for 30 min and > 99% ethanol for 1 h. A thin plastic layer (Agar 100 resin kit, Agar Scientific Ltd) was added to the dishes and incubated for 1 h. The plastic was poured off and a new plastic layer was added onto the dishes for incubation overnight in a desiccator. Next, the plastic was heated to enable its removal after which a new thicker plastic layer was added before another incubation for 1 h in a desiccator. Cells were covered with 3 mm plastic and polymerized in the oven at 60 °C for 48 h. Embedded cells were sectioned by using a Leica ultracut UTC ultrotome (Rowaco AB) and visualized with a Tecnai G2 transmission electron microscope (FEI company) with an ORIUS SC200 CCD camera and Gatan Digital Micrograph software (both from Gatan Inc.).

### Cell lysis

Cell lysis was performed as described previously^19^. In short, cell culture medium was thoroughly removed from the dishes and the cells were lysed in ice-cold lysis buffer (20 mM Tris pH 7.5, 0.5% Triton X-100, 0.5% deoxycholic acid, 150 mM NaCl, 10 mM EDTA, 30 mM NaPyro), supplemented with a protease inhibitor cocktail (ThermoScientific). The lysates were transferred to protein LoBind tubes (Eppendorf) and incubated for 30 min on ice prior to centrifugation at 10,000×*g* for 10 min at 4 °C. The supernatants were transferred to new tubes and stored at − 70 °C until analysis.

### Western blot analysis

Western blot analysis was performed as described previously^19^. In short, protein concentrations of the total cell lysates were measured with Pierce BCA protein kit (ThermoFisher Scientific), according to the manufacturer’s instructions. A total of 18 µg protein was mixed with Bolt LDS Sample buffer and Sample Reducing agent (both from ThermoFisher Scientific) and incubated for 5 min at 95 °C to denature the proteins. Samples were loaded on a Bolt 4–12% Bis–Tris plus gel and run in Bolt MES sodium dodecyl sulfate (SDS) running buffer (both from ThermoFisher Scientific) for 22 min at 200 V. Chameleon duo pre-stained protein ladder (Li-Cor) was used for visualization of gel migration, protein size and orientation. Transfer to a PVDF membrane was performed for 1 h at 20 V in Bolt transfer buffer containing 10% methanol, 0.1% Bolt antioxidant (ThermoFisher Scientific) and 0.01% SDS. Blocking of the membrane was performed in 5% bovine serum albumin (BSA, Sigma-Aldrich) in 0.1% tris-buffered saline-Tween (TBS-T) for 1 h on shake at RT, prior to incubation with primary antibody in 0.5% BSA in 0.1% TBS-T O/N at 4 °C. Primary antibodies used in the study were rabbit anti-Synaptophysin (1:20,000, Abcam) and mouse anti-β-actin (β-actin, 1:1,000, Cell Signaling). Following extensive washes in TBS-T, the membrane was incubated with horseradish peroxidase-conjugated (HRP) secondary goat anti-rabbit or goat anti-mouse antibody (1:20,000, Pierce) in 0.5% BSA in 0.1% TBS-T for 1 h on shake at RT. Development of membrane was performed with enhanced chemiluminescence (ECL, GE Healthcare) by using a ChemiDoc XRS with Image Lab Software to visualize and measure the intensity of the immunoreactive bands (Bio-Rad Laboratories).

### Analyses and statistics

All experiments were performed on 3 independent cell cultures, derived from mouse embryos of different pregnant females. A wide-field microscope (Zeiss AxioImager Z1) and Zen 2012 software was used for image acquisition and analysis. Confocal micrographs were taken with a Zeiss LSM 700 confocal microscope. The number of apoptotic, TUNEL + cells and living cells with non-condensed nuclei was manually counted in totally 30 captured images for each treatment. The number of cells with disrupted mitochondrial networks in CellLight Mitochondria-GFP transfected cultures was manually counted and normalized against the total number of transfected, GFP-expressing cells. A minimum of 135 transfected cells per treatment were analyzed. Since the immunocytochemistry data did not meet the assumption of normal distribution with the Shapiro–Wilk’s W-test, Mann–Whitney *U*-test was used for the mitochondrial data (when only two groups were compared, n = 135) and Kruskal Wallis test followed by Dunn’s multiple comparison test was used for the TUNEL data (when 4 groups were compared, n = 30). The Western blot data was analyzed using Mann–Whitney *U*-test (n = 3). All statistical tests were two-tailed. All results are presented in bar charts with mean ± standard deviation. Levels of significance were set to *p < 0.05, **p < 0.01 and ***p < 0.001.

### Ethics approval

All experiments involving animals were approved by the Uppsala County Animal Ethics Board (ethical permit number: C17/13 and 5.8.18-08472/18), following the rules and regulations of the Swedish Animal Welfare Agency, in compliance with the European Communities Council Directive of 22 September 2010 (2010/63/EU).

## Results

### Endocytosis of EVs results in intracellular Aβ deposits in cortical neurons

**Figure 1:**
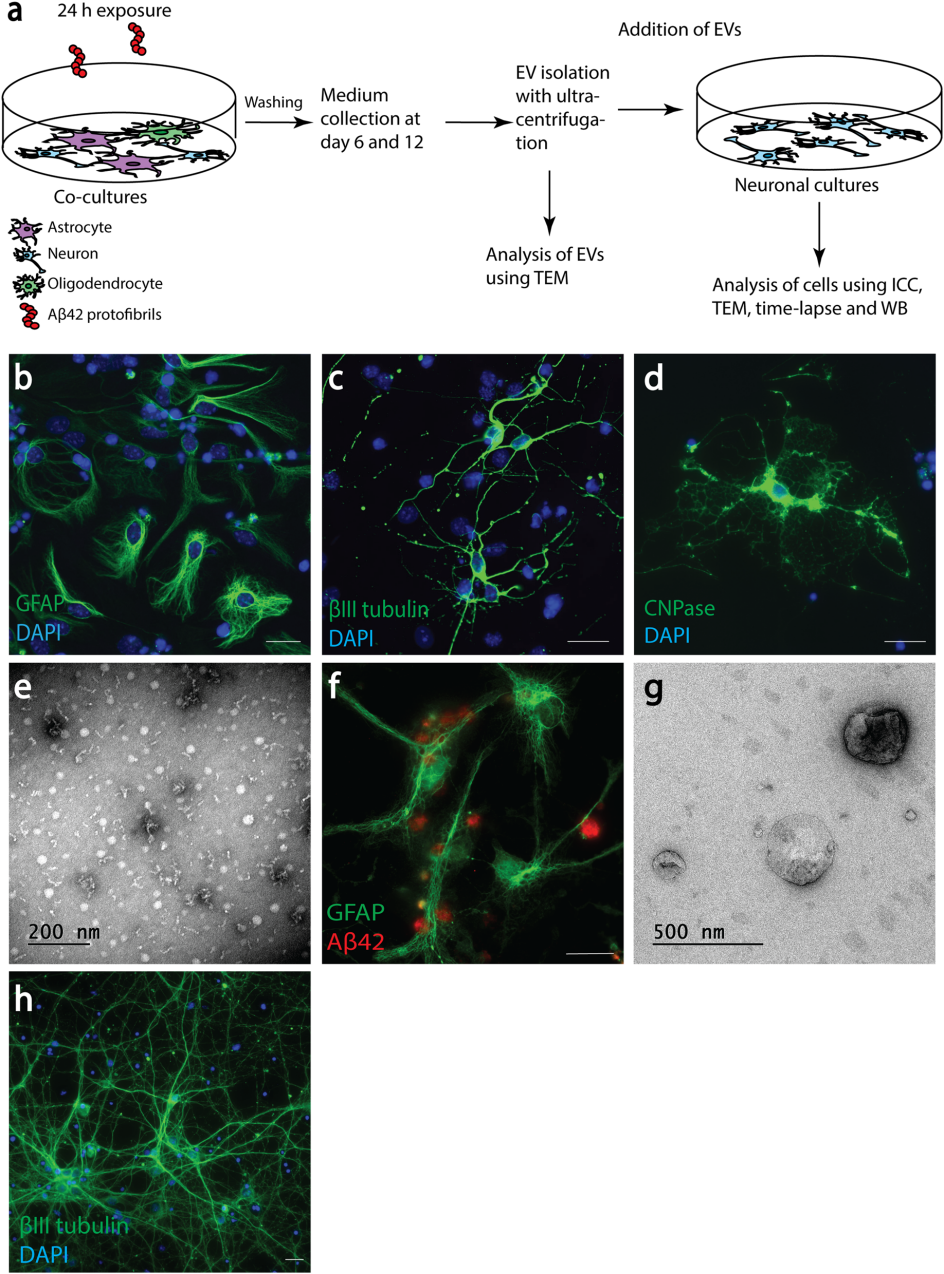
Isolation of EVs from cultures with glial Aβ deposits._._ Schematic experimental set-up for studying EV-mediated neurodegeneration (**a**). Co-cultures of astrocytes (**b**), neurons (**c**) and a few oligodendrocytes (**d**) were exposed to 0.1 µM Aβ_42_ protofibrils (**e**) or left untreated. During the exposure time the astrocytes in the culture ingested and accumulated large amounts of aggregated Aβ (**f**). After 24 h incubation, the cells were washed and incubated in Aβ-free medium for 12 days. Conditioned medium from day 6 and 12 was pooled and ultracentrifuged and successful isolation of EVs was verified by TEM. The EV samples were shown to contain a mixture of exosomes and larger MVs (**g**). Following isolation, the EVs were resuspended in neurobasal medium and added to cortical neuronal cultures (**h**) for 2 or 4 days. Scale bars: (**b**), (**c**), (**d**), (**f**) and (**h**) = 20 µm.

**Figure 2:**
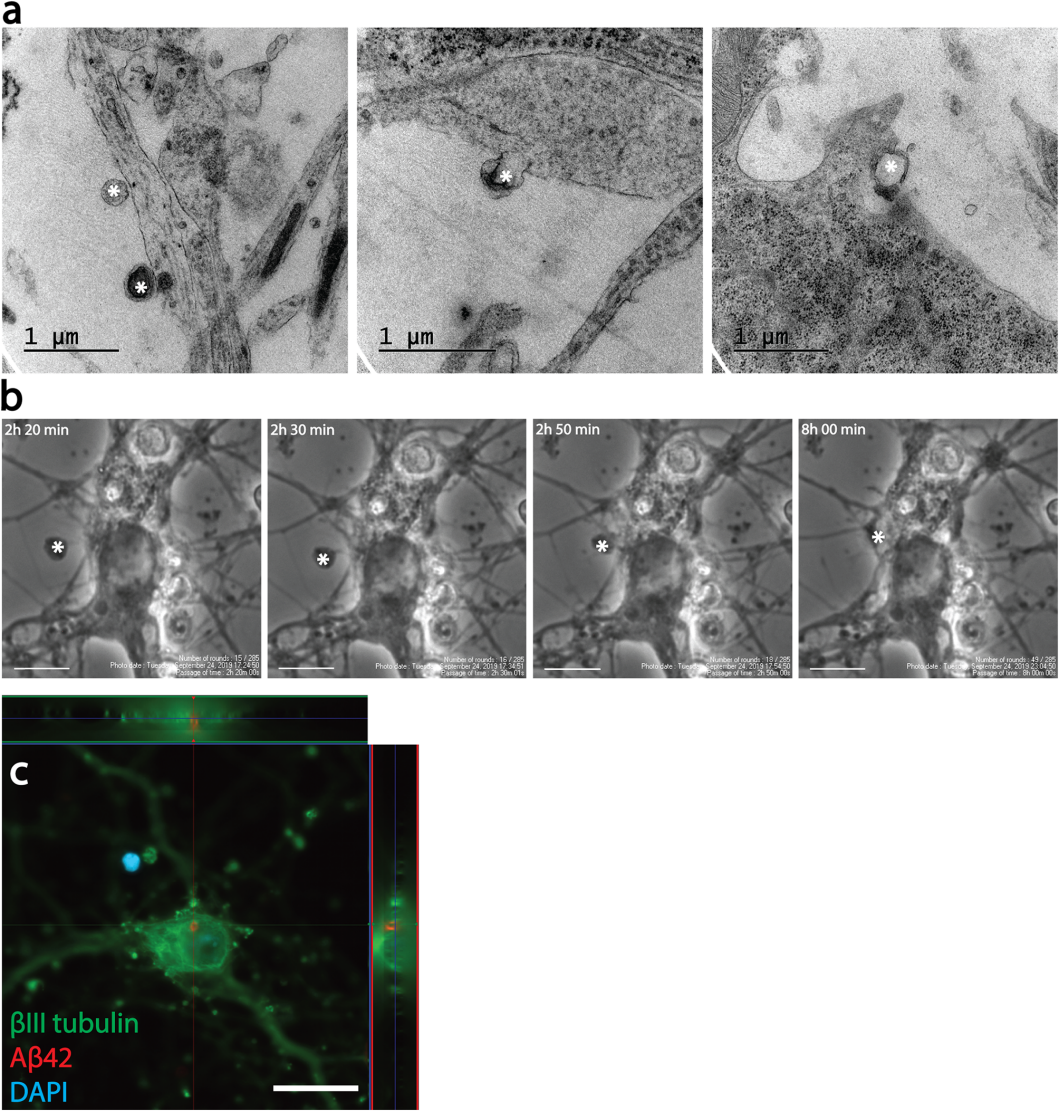
Endocytosis of EVs results in intracellular Aβ deposits in cortical neurons. TEM analysis indicated that EVs attached to and were endocytosed by cortical neurons (asterisks, **a**). The process of EV uptake by the neurons could also be monitored with time-lapse microscopy (asterisk, **b**). Immunocytochemistry followed by confocal microscopy, confirmed intracellular Aβ deposits in Aβ_42_ protofibril-EV exposed neurons (**c**). Scale bars: (**b**) = 10 µm and (**c**) = 20 µm.

In order to study EV-mediated Aβ neurotoxicity, we isolated EVs from cells with intracellular Aβ deposits and added these to cortical neurons (Fig. 1a). More specifically, co-cultures, containing mainly astrocytes (Fig. 1b), but also neurons (Fig. 1c) and a few oligodendrocytes (Fig. 1d) were exposed to Aβ_42_ protofibrils for 24 h (Fig. 1e) or were left untreated. Similar to our previous findings^8,19,25^, the astrocytes in the co-culture ingested large amounts of Aβ_42_ protofibrils, which accumulated intracellularly in lysosomal compartments (Fig. 1f). Following Aβ_42_ protofibril exposure, the co-cultures were cultured without Aβ for 12 days and EVs were isolated from the conditioned medium. TEM analysis of the EV preparation confirmed the presence of exosomes and larger MVs (Fig. 1g). Cortical neurons (Fig. 1h) were exposed to EVs for 2 or 4 days, prior to analysis. TEM analysis and time-lapse microscopy indicated that the added EVs attached to and were endocytosed by the neurons (Fig. 2a,b). Immunocytochemistry of neuronal cultures followed by confocal microscopy confirmed that intracellular Aβ deposits were present in the Aβ_42_ protofibril-EV exposed neurons (Fig. 2c).

### EVs isolated from Aβ_42_ protofibril exposed co-cultures induce apoptosis of cortical neurons

**Figure 3:**
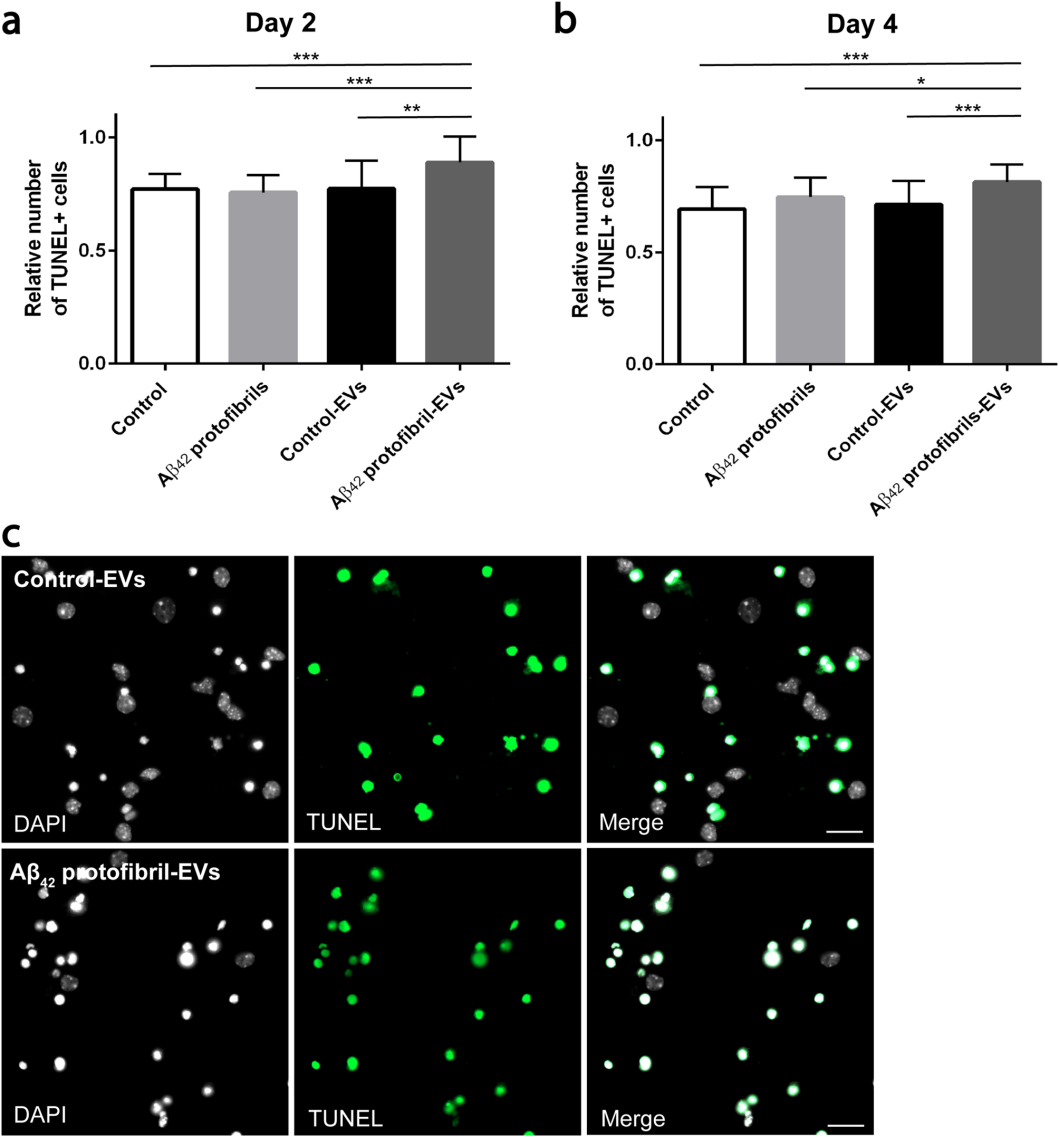
Aβ_42_ protofibril-EVs induce apoptosis of cortical neurons. The relative number of TUNEL + neurons was analyzed 2 and 4 days after EV addition to verify the number of apoptotic cells. Parallel neuronal cultures received medium only (control) or Aβ_42_ protofibrils. There was a significant increase in apoptotic neurons in cultures exposed to Aβ_42_ protofibril-EVs at day 2 (**a**) and day 4 (**b**), compared to neurons that received control-EVs, control medium or medium with Aβ_42_ protofibrils only (*p < 0.05, **p < 0.01 and ***p < 0.001). Representative images of the TUNEL labeling of neuronal cultures exposed to control-EVs and Aβ_42_ protofibril-EVs (**c**). Scale bar: (**c**) = 20 µm.

To investigate if EVs secreted from Aβ_42_ protofibril exposed neuroglial co-cultures affected the survival of cortical neurons, we labeled the neurons with the apoptotic marker TUNEL and quantified the number of apoptotic cells. The relative number of TUNEL + neurons was analyzed in cell cultures exposed to Aβ_42_ protofibril-EVs, control-EVs or Aβ_42_ protofibrils (no EVs) and in untreated control cultures. There were no differences in the relative number of TUNEL + neurons in cultures exposed to Aβ_42_ protofibrils only and untreated controls. Moreover, control-EVs did not affect neuronal survival, compared to the untreated control cultures (Fig. 3a). However, Aβ_42_ protofibril-EVs significantly increased the number of TUNEL + neurons, compared to control-EVs at both day 2 (p < 0.001, Fig. 3a) and day 4 (p < 0.001, Fig. 3b). Representative images of the TUNEL labeling in cultures exposed to control-EVs and Aβ_42_ protofibril-EVs are shown in Fig. 3c. Taken together, the TUNEL analysis indicated a decreased neuronal survival upon exposure to Aβ_42_ protofibril-EVs.

### Aβ_42_ protofibril-EVs cause severe mitochondrial stress in cortical neurons

**Figure 4:**
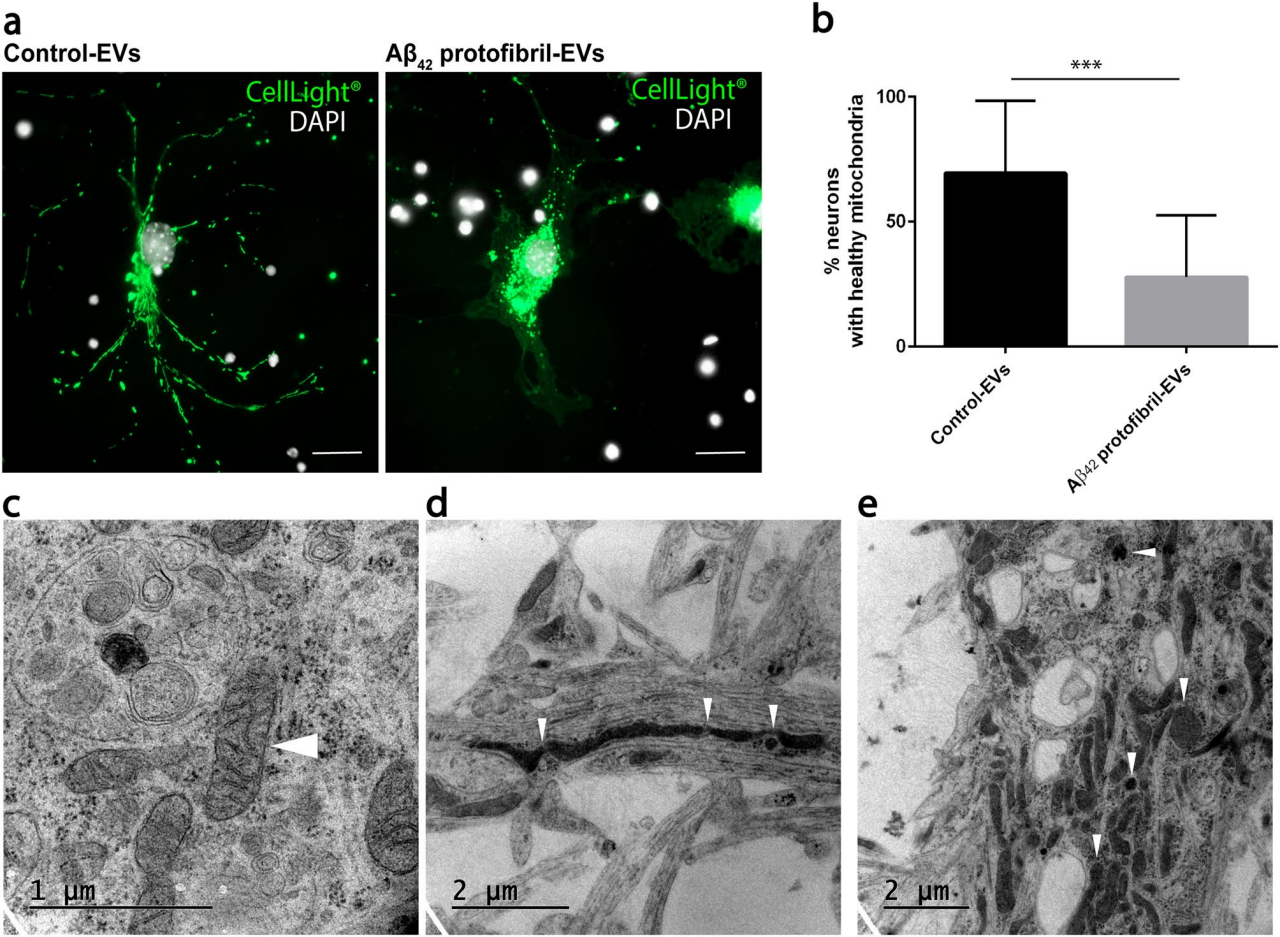
Aβ_42_ protofibril-EV exposure induces mitochondrial stress in cortical neurons. Neurons were transfected with CellLight Mitochondria-GFP 24 h prior to fixation (at day 3), targeting cellular mitochondria. In cultures exposed to control-EVs the neurons displayed an elongated, branched mitochondrial network (healthy), while neurons exposed to Aβ_42_ protofibril-EVs frequently displayed a disrupted mitochondrial network and mitochondrial swelling (unhealthy) (**a**). Quantification revealed a significant decrease in the percentage of healthy mitochondria in neuronal cultures exposed to Aβ_42_ protofibril-EVs, compared to control-EVs (***p < 0.001) (**b**). TEM analysis of neurons exposed to control-EVs verified that their mitochondria appeared healthy (white arrow head) (**c**), whereas Aβ_42_ protofibril-EV exposed neurons had abnormally large, swollen and disrupted mitochondria (white arrow heads) (**d**,**e**). Scale bar: (**a**) = 20 µm.

Since we observed an increased cell death in neuronal cultures following exposure to Aβ_42_ protofibril-EVs, we next sought to investigate the health status of living neurons in the cultures. To examine whether Aβ_42_ protofibril-EV exposed neurons have a disrupted energy metabolism, neurons were transfected with CellLight Mitochondria-GFP 24 h prior to fixation. Neurons that received control-EVs displayed elongated, branched, healthy mitochondrial networks throughout the cells (Fig. 4a). However, in Aβ_42_ protofibril-EV exposed cultures, many neurons displayed a disrupted, unhealthy mitochondrial network and mitochondrial swelling (Fig. 4a). The percentage of neurons with healthy mitochondria, was quantified in relation to the total number of transfected cells. There was a significant decrease in the percentage of healthy mitochondria in cultures exposed to Aβ_42_ protofibril-EVs, compared to control-EVs (p < 0.001, Fig. 4b). The morphology of mitochondria was further visualized by using TEM. The results verified that neurons exposed to control-EVs exhibited healthy mitochondria with clear inner cristae (Fig. 4c), while mitochondria in neurons exposed to Aβ_42_ protofibril-EVs were clearly affected, showing loss of structure of the outer membrane as well as the inner cristae, and abnormal size compared to the control-EV exposed neurons (Fig. 4d,e).

### Impaired synaptic organization in Aβ_42_ protofibril-EV exposed cortical neurons

**Figure 5:**
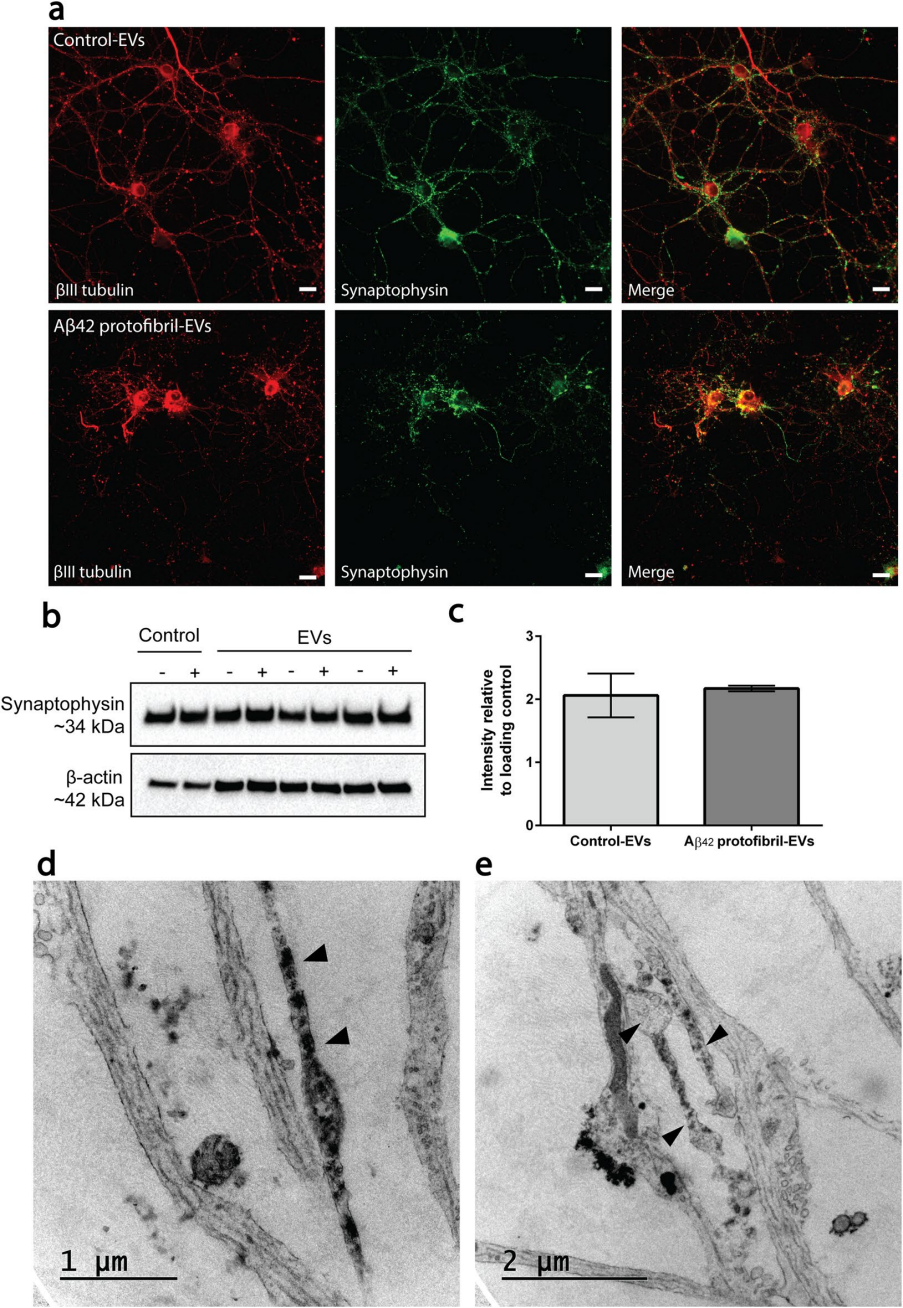
Aβ_42_ protofibril-EV exposure results in synaptic loss in cortical neurons. Neuronal cultures were co-immunostained with the neuronal-specific marker βIII tubulin and the synaptic vesicle marker synaptophysin. In cultures exposed to control-EVs, the neurons displayed a large network of synaptophysin-positive processes at day 2. In contrast, neurons exposed to Aβ_42_ protofibril-EVs displayed a severely disrupted network (**a**). Intracellular synaptophysin levels, measured by Western blot analysis for 3 independent neuronal cell culture batches, showed no differences between Aβ_42_ protofibril-EV exposed neurons (+) and neurons that received control-EVs (−), control medium (−) Aβ_42_ protofibrils (+), respectively (**b**,**c**). TEM analysis of neuronal processes in cultures exposed to Aβ_42_ protofibril-EVs exhibited pathological signs of abnormal swelling and accumulation of filled vesicles (**d**,**e**). Scale bar: (**a**) = 20 µm.

Next, we aimed to study if Aβ_42_ protofibril-EVs induce synaptic alterations. For this purpose, neurons were stained with the neuronal-specific marker βIII tubulin and the synaptic vesicle marker synaptophysin. Neurons that received control-EVs displayed long axonal and dendritic networks at day 2 (Fig. 5a). In contrast, addition of Aβ_42_ protofibril-EVs resulted in severe synaptic loss and disrupted dendrites (Fig. 5a). Western blot analysis of the lysed neurons revealed that intracellular synaptophysin levels were unaffected in all neuronal cultures at day 2 (Fig. 5b; Supplementary Fig. 1). This was confirmed by intensity measurements of synaptophysin immunoreactive bands, analyzed relative to the loading control β-actin (Fig. 5c). To further investigate the effect of Aβ_42_ protofibril-EVs on the neuronal network, we performed TEM. The Aβ_42_ protofibril-EV exposed neurons displayed distinct pathology, including accumulation of filled vesicles and vacuoles (Fig. 5d), and abnormal swelling of processes (Fig. 5e). Taken together, our data suggest that the synaptophysin levels were not altered in response to Aβ_42_ protofibril-EVs, but its structural organization within the neurons was markedly affected.

### Aβ_42_ protofibril-EVs induce enlarged vacuoles and waste accumulation in cortical neurons

**Figure 6:**
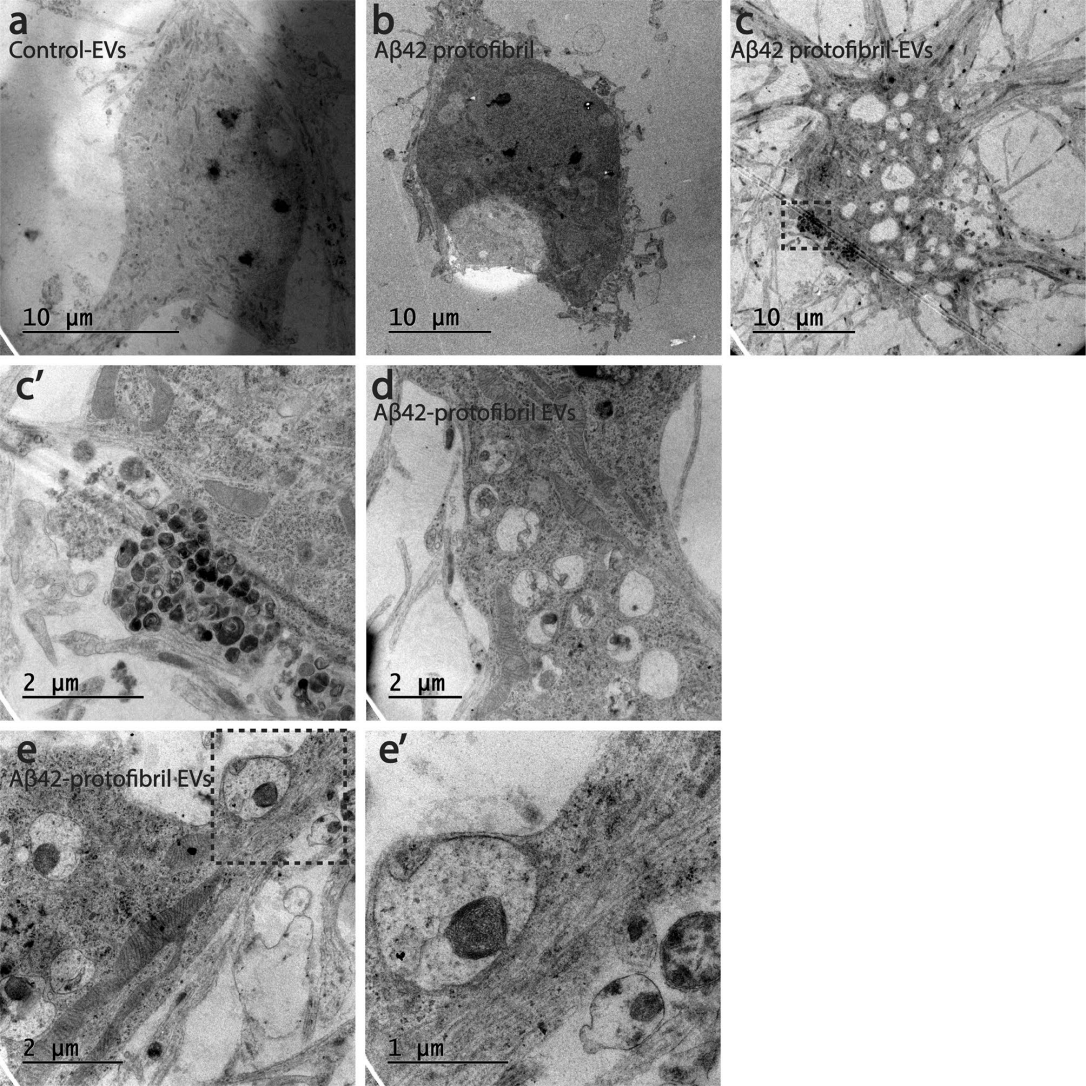
Addition of Aβ_42_ protofibril-EVs induces vacuole formation in neuronal cell bodies. TEM analysis demonstrated that the cell bodies of neurons exposed to control-EVs (**a**) or free-floating Aβ_42_ protofibrils (**b**) were compact and healthy, while Aβ_42_ protofibril-EVs induced massive vacuole formation in the neuronal cell bodies (**c**). A higher magnification of the dashed area in c display a group of vesicles filled with debris (**c’**). Also, some of the larger vacuoles were filled with various forms of waste (**d**), including condensed mitochondria (**e**). A higher magnification of the dashed area is shown in (**e’**).

In order to investigate subcellular changes in the neurons following exposure to Aβ_42_ protofibril-EVs, we performed additional TEM analysis. In comparison to cortical neurons treated with control-EVs (Fig. 6a) or free-floating Aβ_42_ protofibrils (Fig. 6b), Aβ_42_ protofibril-EV exposed neurons displayed multiple enlarged vacuoles. The vacuoles appeared to be empty or filled with diverse waste material (Fig. 6c,d). For example, the neurons contained many dense vacuoles (Fig. 6c) and lysosomal or endosomal compartments containing condensed mitochondria (Fig. 6e). The pathological vacuolization in combination with the high load of waste indicates that the neurons’ degradation capacity was severely affected by the exposure to Aβ_42_ protofibril-EVs.

### Lamellar body formation is frequently present in EV exposed cortical neurons

**Figure 7:**
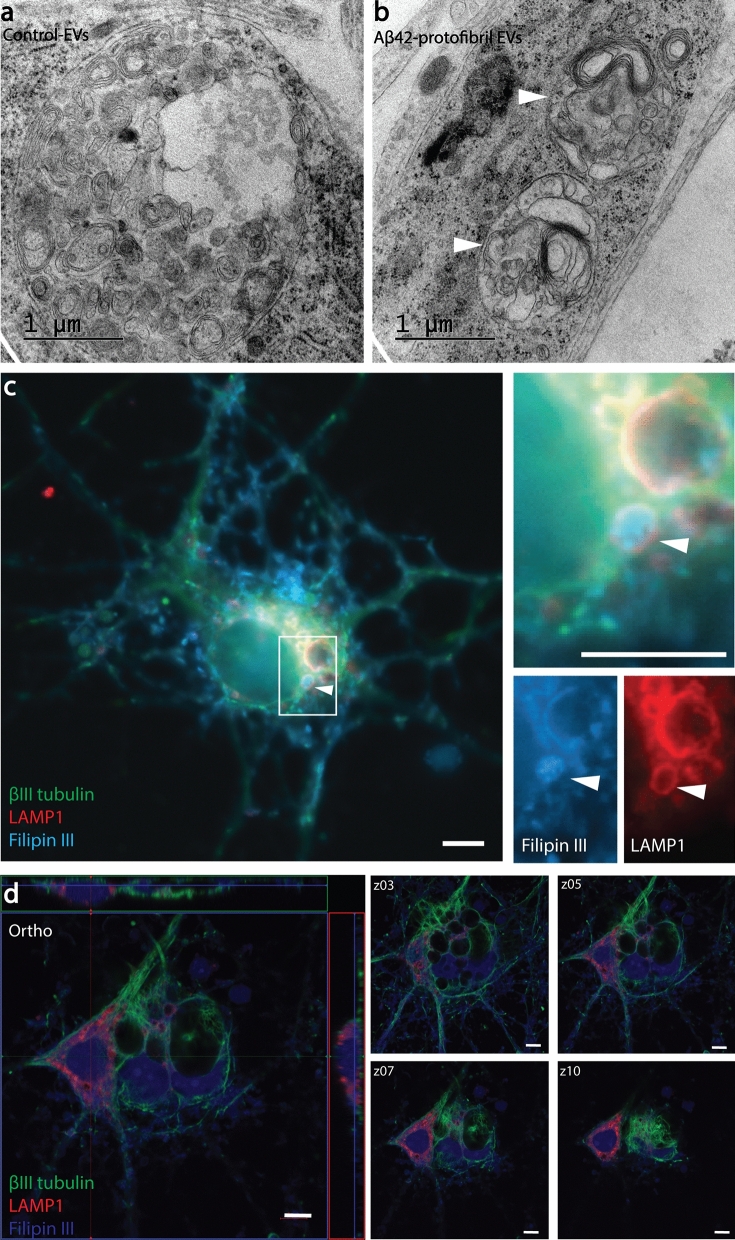
Addition of EVs induces the formation of lamellar inclusions. Vesicles filled with lipid membranes were observed in TEM analysis of cortical neurons exposed to both control-EVs and Aβ_42_ protofibril-EVs (arrowheads, **a**, **b**). Immunocytochemical stainings, followed by fluorescence microscopy (**c**) and confocal microscopy (**d**) demonstrated that cholesterol deposits in neurons exposed to Aβ_42_ protofibril-EVs were situated in LAMP-1 positive compartments, verifying the presence of lamellar inclusions. Scale bar: (**c**) = 5 µm and (**d**) = 20 µm.

Interestingly, neurons that received either Aβ_42_ protofibril-EVs or control-EVs displayed multilamellar bodies (Fig. 7a,b). Multilamellar bodies are structures with multiple membranes, generated in lysosomal compartments, primarily consisting of undegraded phospholipids and cholesterol. In various disease conditions, the appearance of multilamellar bodies has been shown to be closely related to excessive cholesterol accumulation^26,27^. Hence, we next stained the neuronal cultures with the fluorescent cholesterol-binding dye filipin III. Co-stainings using specific antibodies against the lysosomal protein LAMP1, followed by fluorescence and confocal microscopy, confirmed that multilamellar bodies in Aβ_42_ protofibril-EV exposed cortical neurons consisted of cholesterol deposits in lysosomal compartments (Fig. 7c,d). A zoomed out image of the stained cell culture shown is shown in Supplementary Fig. 2.

## Discussion

Knowledge of the exact mechanisms behind neuronal atrophy in AD, as well as information about other cell types’ contribution to the neurodegeneration process is still very limited. To enable the development of effective therapeutic interventions for AD patients, a better understanding of the basic cellular mechanisms underlying the disease progression is crucial. In the present study we are clarifying the impact of EVs in Aβ-induced neurotoxicity. Taken together, our results show that EVs from Aβ_42_ protofibril exposed neuroglial co-cultures induce mitochondrial disturbances, synaptic loss and neuronal cell death in cortical neurons, indicating that EVs could play a critical role in the neurodegeneration process of AD.

Many lines of evidence suggest that Aβ pathology and inflammation are closely associated. Both reactive astrocytes and microglia are frequently found in the AD brain, especially around the plaques^28^, but their impact on AD progression remains elusive. While the formation of a glial capsule around the Aβ deposits may have a protective effect on the brain tissue by isolating the toxic Aβ species, the glial cells may also spread neurotoxic products to surrounding cells, for example via EVs^8,29,30^.

The aim of the present study was to elucidate in which ways EVs, released from the major brain cell types, are involved in Aβ-mediated neurodegeneration. For this purpose, EVs isolated from Aβ_42_ protofibril exposed neuroglial co-cultures were added to cortical neurons. Subsequently the viability of the neurons was analyzed, as well as their health status with respect to morphology, synapses and mitochondria. Astrocytes are the most abundant glial cell type in the human brain and they are crucial for maintaining tissue homeostasis^31^. Astrocytes have a wide variety of functions, including metabolic support of neurons, modification of synapse signaling, recycling of neurotransmitters, blood–brain barrier regulation and glymphatic clearance^31–33^. In the pathological brain, astrocytes are converted to a reactive, inflammatory state^34^. The impact of reactive astrocytes on AD progression is probably largely due to their uptake and release of substances from the microenvironment that they share with neurons. Astrocytes effectively engulf dead cells, damaged synapses and protein aggregates^8,35–39^. Interestingly, reactive astrocytes have been shown to be more efficient than microglia in taking up Aβ^40^. The fact that reactive astrocytes with high Aβ load are frequently found in the human AD brain, further confirms the importance of astrocytes in Aβ pathology^35,38^.

EVs are a heterogeneous group of secretory vesicles that are known to be crucial for cell-to-cell communication^41^. It has been shown that EVs can promote neuronal progression and regeneration, but also contribute to neurodegenerative diseases^42^. In the present investigation, endocytosis of EVs by cortical neurons was verified by time-lapse microscopy and TEM. Moreover, EV-mediated spreading of Aβ deposits was demonstrated, using immunocytochemistry and confocal imaging. The majority of the cells in the co-cultures, from which the EVs were isolated, were astrocytes that displayed large intracellular Aβ deposits. We have previously shown that astrocytes ingest very large amounts of Aβ_42_ protofibrils that are accumulated intracellularly in lysosomal compartments, rather than degraded^8^. Interestingly, the Aβ_42_ deposits co-localize with LAMP-1, but not with LysoTracker labelling, indicating that the Aβ_42_ containing lysosomes are not fully mature^8^. Importantly, the inability of astrocytes to clear Aβ leads to increased release of neurotoxic N-terminally truncated forms of Aβ in EVs^8^. It is likely that the secretion of EVs may serve as a clearing mechanism, in which the astrocytes try to get rid of toxic material, such as aggregated proteins that they have failed to degrade. Our previous analyses have further demonstrated that apoE is highly elevated in EVs isolated from Aβ_42_ protofibril exposed co-cultures^19^. In the present study, we show that Aβ_42_ protofibril exposure of neuroglial co-cultures results in the release of neurotoxic EVs. TUNEL assays demonstrated that neuronal cultures exposed to Aβ_42_ protofibril-EVs contained a significantly increased number of apoptotic nuclei at both 2 and 4 days, compared to cultures exposed to control-EVs. Interestingly, free-floating Aβ_42_ protofibrils (without EVs) were much less toxic than the Aβ_42_ protofibril-EVs. This can be explained by the fact that astrocytes in the co-culture only partly degrade the ingested Aβ_42_ protofibrils and instead secrete a more toxic, truncated form of Aβ aggregates^8^. Since EVs can be internalized by target cells through different pathways, including endocytosis, phagocytosis and macropinocytosis, it is possible that Aβ aggregates that are situated within EVs are more harmful to the neurons, because of their entering route^17^. Neuronal uptake of Aβ packed in EVs is probably more effective than uptake of free-floating aggregates and the ingested Aβ may also end up in different intracellular compartments and thereby induce more damage.

The function of mitochondria has been suggested to be severely compromised in AD. Mitochondria from AD brains have reduced membrane potential, increased permeability and express excessive levels of reactive oxygen species (ROS), the latter known to culminate in oxidative stress and cell damage^43–45^. Mitochondria also have an important role in the regulation of cell death. Upon apoptotic stimuli, mitochondria release several death factors, which may trigger neurodegeneration in AD^46^. Here, we show that Aβ_42_ protofibril-EVs induce mitochondrial disruption in neurons, suggesting that mitochondria are unable to maintain the energy homeostasis of the cell and are thus likely to undergo apoptosis. Quantification of neurons with healthy and unhealthy mitochondria showed that there was a significant decrease in the percentage of healthy mitochondria in neuronal cultures exposed to Aβ_42_ protofibril-EVs, compared to control-EVs. Moreover, TEM analysis verified that neurons exposed to control-EVs exhibited healthy mitochondria, while neurons exposed to Aβ_42_ protofibril-EVs did not. The mitochondria in these neurons were abnormal in size and displayed loss of structure of the outer membrane as well as the inner cristae.

Extensive research has been investigating the synaptic dysfunction in AD. In *post-mortem* human AD brains, a significant loss of important synaptic vesicle proteins, including synaptophysin, SV2 and p65 has been observed, particularly in the neocortex and hippocampus^47,48^. Furthermore, it has been shown that increased levels of Aβ oligomers correlate with the loss of synaptic markers in *post-mortem* frontal cortex samples from AD patients^49^. In the present study, we demonstrate that Aβ_42_ protofibril-EV exposure induces synaptic defects in cortical neurons. Analysis with TEM demonstrated that the Aβ_42_ protofibril-EVs induce axonal swelling and deposition of waste-filled vesicles in the neuronal processes. In addition, immunocytochemistry demonstrated that Aβ_42_ protofibril-EVs caused severe disruption of the neuronal network.

Interestingly, Aβ_42_ protofibril-EV exposed neurons displayed multiple enlarged vacuoles that appeared either empty or filled with diverse waste material, indicating that their degradation capacity was severely affected. Some of the vacuoles had the typical appearance of lipid droplets, but EV exposed neurons also showed the distinct feature of multilamellar bodies. Multilamellar bodies, also called lamellar inclusions, are multi-layered lysosomal organelles that store and secrete lipids as their main function. They can be found in various types of cells in the body and are for example frequent in the lung and epidermis. Interestingly, lamellar bodies also exist in the brain, but only under pathological conditions, such as Niemann-Pick disease, where lysosomal storage is severely affected^26,27^. Immunocytochemistry of the cortical neurons with the cholesterol-binding dye filipin III indicated that the lamellar bodies consisted of cholesterol deposits in lysosomal compartments.

Taken together, our data show that the secretion of EVs from Aβ_42_ protofibril exposed cells induces neuronal dysfunction in several ways, indicating a critical role for EVs in the progression of Aβ-induced pathology. A pathogenic role for EVs in spreading of aggregation-prone proteins has been implicated in various neurodegenerative diseases, including AD^50,51^. However, many questions remain to be answered, including the involvement of various cell types and how to interfere with the spreading. In conclusion, our analysis of the neurotoxic effects of Aβ_42_ protofibril-EVs in terms of synapse alterations, mitochondrial impairment, neuronal swelling, vacuolization, degradation defects and increased apoptosis strongly supports the hypothesis that EVs contribute to Aβ-induced pathology and we encourage further studies aimed to reverse or prevent the negative actions of EVs in order to limit disease propagation.

## Supplementary information

Supplementary Information

## Data Availability

All data is included in the article. Raw data for the mitochondria, TUNEL and Western blot graphs are available from the corresponding author upon request. All data generated or analyzed during this study are included in this article.
