# Supplementary material for: Extracellular vesicles from amyloid-β exposed cell cultures induce severe dysfunction in cortical neurons
Source: Sci Rep. 2020 Nov 12;10:19656. doi: 10.1038/s41598-020-72355-2 (PMC7661699; doi:10.1038/s41598-020-72355-2)
Supplement: Supplementary file 1 — Supplementary Information [file 41598_2020_72355_MOESM1_ESM.pdf]

Extracellular vesicles from amyloid-β exposed cell cultures induce severe dysfunction in cortical neurons

Chiara Beretta[1], Elisabeth Nikitidou[1], Linn Streubel-Gallasch[1], Martin Ingelsson[1], Dag Sehlin[1] and Anna Erlandsson[1]

[1] Department of Public Health & Caring Sciences, Molecular Geriatrics, Rudbeck Laboratory, Uppsala University, Uppsala, Sweden

Corresponding author

Anna Erlandsson

Department of Public Health and Caring Sciences / Molecular Geriatrics,

Rudbeck Laboratory, Uppsala University

SE-751 85 Uppsala, Sweden

E-mail: *anna.erlandsson@pubcare.uu.se*

Co-authors

Chiara Beretta

Department of Public Health and Caring Sciences / Molecular Geriatrics,

Rudbeck Laboratory, Uppsala University

SE-751 85 Uppsala, Sweden

E-mail: *chiara.beretta@pubcare.uu.se*

Elisabeth Nikitidou

Department of Public Health and Caring Sciences / Molecular Geriatrics,

Rudbeck Laboratory, Uppsala University

SE-751 85 Uppsala, Sweden

E-mail: *elisabeth.nikitidou@telia.com*

Linn Streubel-Gallasch

Department of Public Health and Caring Sciences / Molecular Geriatrics,

Rudbeck Laboratory, Uppsala University

SE-751 85 Uppsala, Sweden

E-mail: *linn.streubel-gallasch@pubcare.uu.se*

Martin Ingelsson

Department of Public Health and Caring Sciences / Molecular Geriatrics,

Rudbeck Laboratory, Uppsala University

SE-751 85 Uppsala, Sweden

E-mail: *martin.ingelsson@pubcare.uu.se*

Dag Sehlin

Department of Public Health and Caring Sciences / Molecular Geriatrics,

Rudbeck Laboratory, Uppsala University

SE-751 85 Uppsala, Sweden

E-mail: *dag.sehlin@pubcare.uu.se*

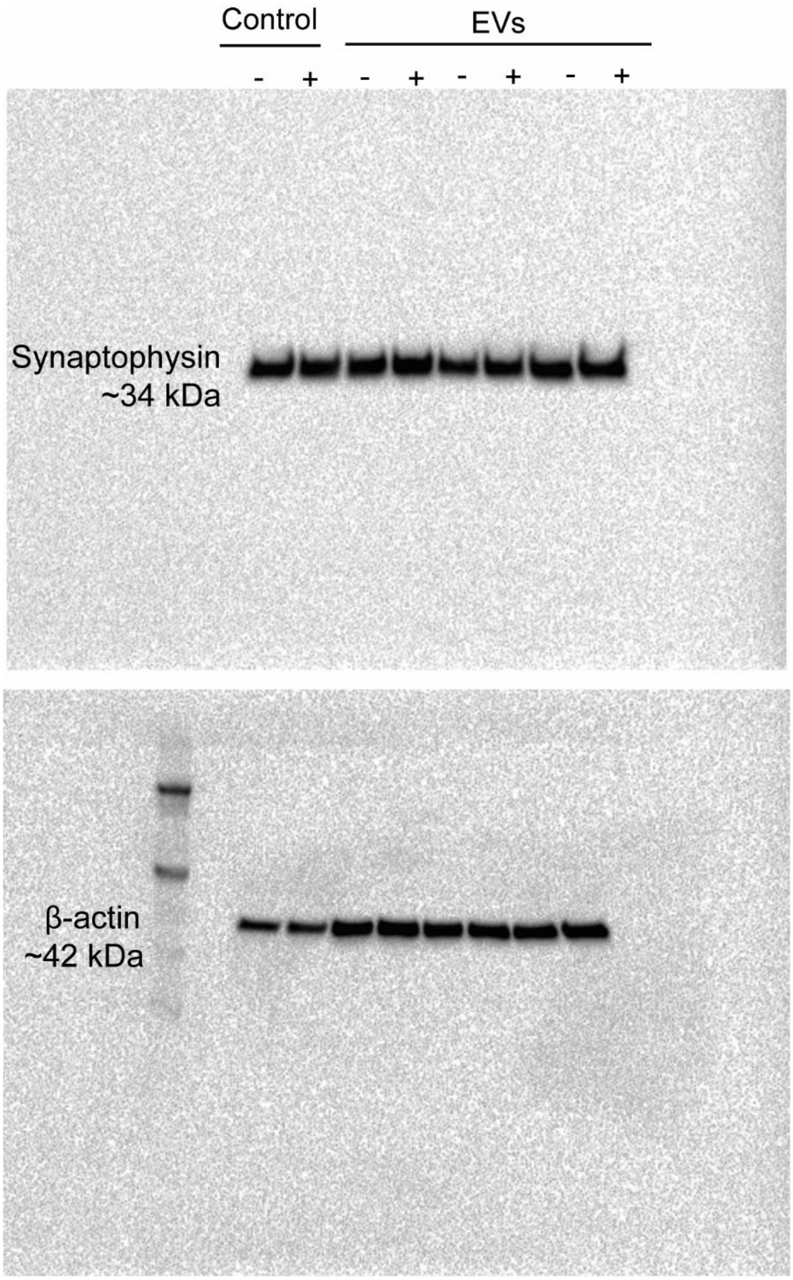

Supplementary Figure 1.

Unprocessed, full membrane images of the Western blot analysis of synaptophysin and β-actin shown in Figure 5.

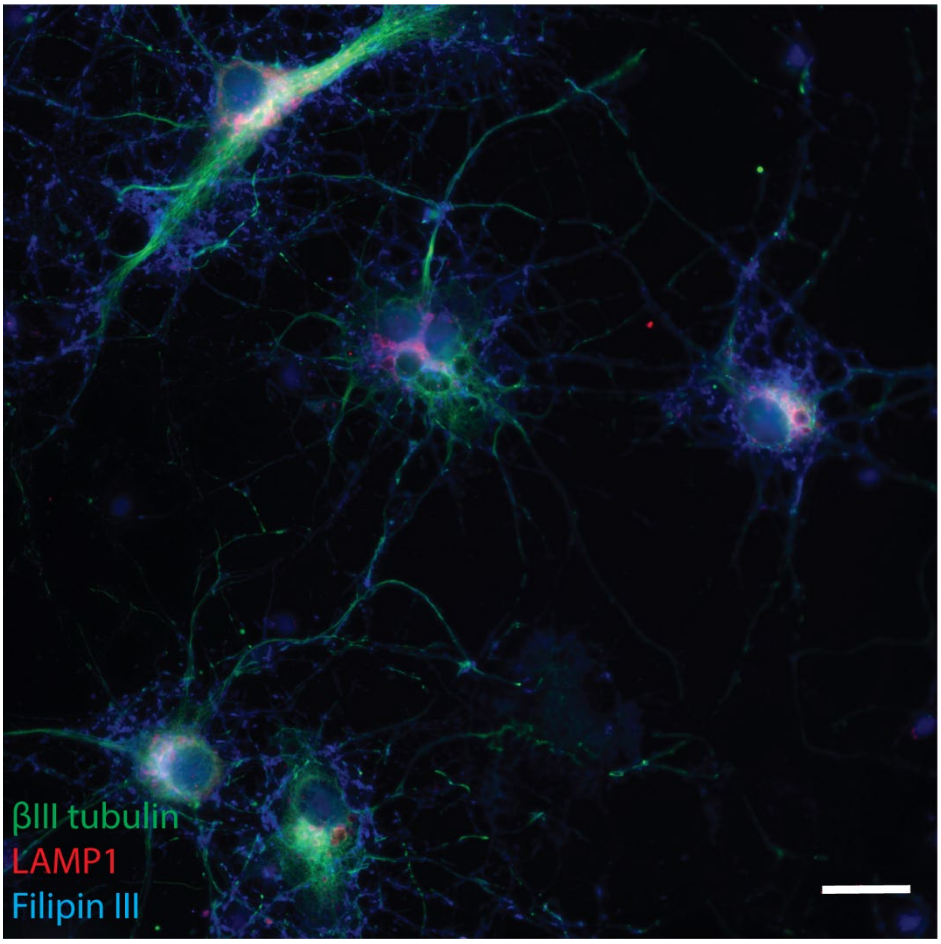

Supplementary Figure 2.

A zoomed out image of the stained cell culture shown in Figure 7 c, illustrating that the membranes of the neurons are positive for the cholesterol-binding dye filipin III. Scale bar: c=20 μm.
